# Clinical evidence and rationale of mesoglycan to treat chronic venous disease and hemorrhoidal disease: a narrative review

**DOI:** 10.1007/s13304-024-01776-9

**Published:** 2024-02-14

**Authors:** Gaetano Gallo, Arcangelo Picciariello, Antonella Tufano, Giuseppe Camporese

**Affiliations:** 1 Department of Surgery, Sapienza University of Rome, Rome, Italy; 2 Department of Experimental Medicine, University of Salento, Lecce, Italy; 3 Department of Clinical Medicine and Surgery, University of Naples Federico II, Naples, Italy; 4 Department of Internal Medicine, Padua University Hospital, Padua, Italy

**Keywords:** Chronic venous disease, Glycocalyx, Hemorrhoidal disease, Mesoglycan

## Abstract

Chronic venous disease (CVD) and hemorrhoidal disease (HD) are among the most common vascular diseases in the world, with CVD affecting 22–41% of the population in Europe and HD having a point prevalence of 11–39%. The burden is substantial in terms of the effect of symptoms on patients’ health-related quality of life (HRQoL) and direct/indirect medical costs. Treatment begins with lifestyle changes, compression in CVD and topical therapies in HD, and escalates as needed through oral therapies first and eventually to surgery for severe disease. CVD and HD share etiological features and pathological changes affecting the structure and function of the tissue extracellular matrix. Mesoglycan, a natural glycosaminoglycan (GAG) preparation composed primarily of heparan sulfate and dermatan sulfate, has been demonstrated to positively impact the underlying causes of CVD and HD, regenerating the glycocalyx and restoring endothelial function, in addition to having antithrombotic, profibrinolytic, anti-inflammatory, antiedema and wound-healing effects. In clinical trials, oral mesoglycan reduced the severity of CVD signs and symptoms, improved HRQoL, and accelerated ulcer healing. In patients with HD, mesoglycan significantly reduced the severity of signs and symptoms and the risk of rectal bleeding. In patients undergoing excisional hemorrhoidectomy, adding mesoglycan to standard postoperative care reduced pain, improved HRQoL, reduced incidence of thrombosis, and facilitated an earlier return to normal activities/work, compared with standard postoperative care alone. The clinical effects of mesoglycan in patients with CVD or HD are consistent with the agent’s known mechanisms of action.

**Supplementary Information:**

The online version contains supplementary material available at 10.1007/s13304-024-01776-9.

## Introduction

Chronic venous disease (CVD) and hemorrhoidal disease (HD) are among the most common vascular diseases in the world. CVD is estimated to affect approximately 22–41% of the population in Europe [1, 2], while HD has a point prevalence of 11–39% [3, 4]. Although these venous conditions do not cause acute catastrophic vascular events, as arterial disease can, they nevertheless carry a substantial economic burden [5, 6] and can negatively affect the individual’s health-related quality of life (HRQoL) [7, 8].

While there is no evidence that CVD and HD are intrinsically linked, the pathophysiological changes in both affect the structure and function of the extracellular matrix, as well as the integrity of supporting tissues [9–11]. In both conditions, the usual approach to treatment is to start conservatively, with lifestyle changes, compression in CVD and topical therapies in HD, and escalate as needed through oral therapies first and eventually to surgery for severe disease with a significant impact on HRQoL [12–18].

Mesoglycan, a porcine intestinal mucosa extract, is a natural preparation of glycosaminoglycans (GAGs) consisting mainly of heparan sulfate (47.5%) and dermatan sulfate (35.5%), and to a lesser extent, slow-moving heparin (8.5%) and chondroitin sulfate (8.5%) that has been used for several years to treat CVD and HD [19–21]. While three reviews that mention the use of mesoglycan have been published in the last decade [22–24], no review has focused specifically on the drug. The aim of this narrative review is to examine the clinical effects of mesoglycan in patients with CVD or HD, with a focus on how the agent’s mechanism of action can favorably modify the underlying pathophysiological processes in these two conditions.

## Methods

A literature search of PubMed was undertaken on 17 April 2023 to identify studies of mesoglycan in patients with CVD or HD. These results were reviewed manually to identify the best quality available evidence. The literature search was supplemented by ad hoc searches on specific topics as needed and by literature known to the authors that may be relevant to the topic, but not indexed on PubMed.

### Chronic venous disease

CVD is typically classified using the Clinical–Etiological–Anatomical–Pathological (CEAP) system, where the C-class defines the visible signs (Supplementary Table S1) [25]. The symptoms of CVD include aching, heaviness, cramps, itching/tingling in the legs, and restless legs [1, 9, 15]. Affected individuals often develop spider veins, varicose veins, edema, hyperpigmentation, and potentially leg ulcers [1, 9].

The risk of CVD is increased in genetically susceptible individuals, older people, women, those receiving estrogen therapy, people who are overweight or obese, pregnant women, patients with a history of superficial venous thrombosis (SVT) or leg injury, and those with a sedentary lifestyle or long-term immobilization [26, 27].

Due to its progressive nature [13], worsening CVD is associated with a deterioration in the affected individual’s HRQoL [28]. It may also be associated with several potentially serious complications, such as venous ulcers, cellulitis, thromboembolic events, and post-thrombotic syndrome [14, 15].

### Pathophysiology

**Fig. 1:**
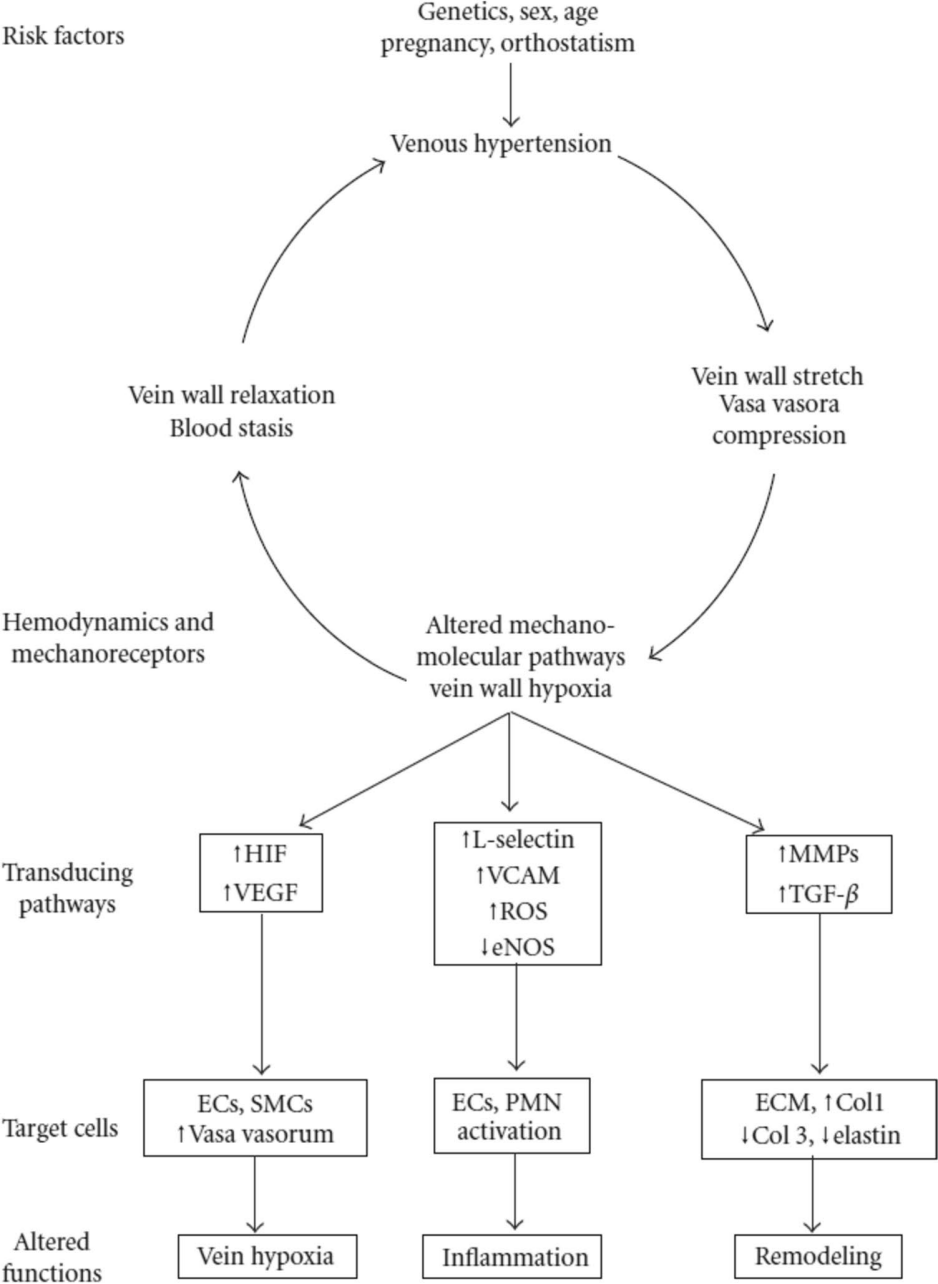
Signaling pathways involved in the pathogenesis of chronic venous disease [30]. *Col* collagen, *EC* endothelial cell, *ECM* extracellular matrix, *eNOS* endothelial nitric oxide synthase, *HIF* hypoxia-inducible factor, *MMP* matrix metalloproteinase, *PMN* polymorphonuclear leukocyte, *ROS* reactive oxygen species, *SMC* smooth muscle cell, *TGF-β* transforming growth factor-beta, *VCAM* vascular cellular adhesion molecule, *VEGF* vascular endothelial growth factor. Reproduced from Atta HM. Int J Vasc Med Int J Vasc Med 2012:538627, 10.1155/2012/538627 under a CC BY 3.0 DEED license (https://creativecommons.org/licenses/by/3.0/

Venous hypertension and altered drainage, with venous valve incompetence and reflux, are associated with the development of CVD [9, 29]. CVD usually starts in the superficial veins of the lower leg, which lack the external support of surrounding calf muscles to help maintain venous return [27]. The increase in venous pressure and venous dilation reduces the shear stress on the endothelial surface of the vein [27]. Reflux contributes to the increase in venous pressure and causes turbulent flow, which is detected by mechano-sensors and endothelial cells, triggering a complex biological response (Fig. 1) [14, 29, 30].

The glycocalyx has a key role in this process. The glycocalyx is a nexus layer composed of soluble components and membrane-bound glycoproteins and GAGs, which overlays the luminal surface of the endothelium and helps to maintain homeostasis [31]. Membrane-bound GAGs (principally heparan sulfate, dermatan sulfate, and chondroitin sulfate) form the structure of the glycocalyx, providing a grid to hold the soluble components on the surface of the endothelial cell [31]. These soluble components include hyaluronic acid, thrombomodulin, extracellular superoxide dismutase, albumin, and antithrombin III [31, 32]. Of the GAGs in the vasculature, heparan sulfate is the most abundant comprising between 50 and 90% of the total GAG content [33].

The integrity of the glycocalyx is essential for endothelial cells' performance and vascular homeostasis. This layer is continuously damaged and replenished by soluble components from plasma or from endothelial cells [31, 32]. Shear stress may damage the glycocalyx, disrupting the labile homeostasis that is usually maintained within veins (Table 1) [32, 34]. Damage to the glycocalyx exposes adhesion molecules, such as intercellular adhesion molecule 1 (ICAM-1) and vascular cell adhesion molecule 1 (VCAM-1), resulting in leukocyte adhesion [34]. This activates the release of proinflammatory cytokines and chemokines, including interleukin (IL)-1β, IL-6, IL-8, L-selectin and tumor necrosis factor (TNF)-α, which leads to local inflammation and accelerated leukocyte adhesion [34].

**Table 1:** Functions of the endothelial glycocalyx

Anti-inflammatory properties	Controls platelet and leucocyte adhesion, and hemostatic and inflammatory responses
Barrier function and vascular leakage	Controls fluid exchange and balance, protein forces, and water movement
Vascular tone regulation	Releases nitric oxide, maintains vasodilatation and vasoconstriction balance, and inhibits and promotes proliferation and migration of smooth muscle cells
Antithrombotic and fibrinolytic activity	Maintains blood rheology, and balances coagulation and fibrinolysis counteracting platelet activation and aggregation by the release of antithrombin III, t-PA, and vWF
Antioxidant properties	Protects the endothelium by binding enzymes that metabolize oxygen radicals, such as extracellular superoxide dismutase

*t-PA* tissue plasminogen activator, *vWF* von Willebrand factor

When the glycocalyx is damaged, nitric oxide (NO) release is impaired, affecting the vein’s normal contractile response to changes in shear stress [31]. Moreover, the damage to the glycocalyx also shifts the balance from an anticoagulant to procoagulant environment at the endothelial surface [31, 32].

Endothelial dysfunction and the inflammatory response promote structural changes in the vein wall [35]. Endothelial inflammation stimulates the proliferation of smooth muscle cells in the intimal layer. At the same time, fibroblasts produce matrix metalloproteinases (MMPs) [13], resulting in an imbalance between tissue inhibitors of MMPs (TIMPs) and MMPs in favor of MMPs, and causing degradation of collagen and elastin bundles in the medial layer of the vein, as well as breakdown of the extracellular matrix (ECM) [35].

These mechanisms also contribute to vein damage and valve degradation, which exacerbate the hemodynamic changes, eventually resulting in the signs and symptoms of CVD, including the development of varicose veins and the appearance of skin changes [35].

Furthermore, the above-mentioned endothelial dysfunction and inflammatory response observed in CVD are also observed in cardiovascular disease [2]. Indeed, CVD and cardiovascular disease share risk factors such as age, obesity, female sex, smoking, family history, and prior venous thrombosis [36], providing further support for a close pathophysiological relationship between CVD and cardiovascular disease. It has been reported that “the legs are a pathway to the heart” and that pathophysiological improvements of endothelial dysfunction, inflammation, and thrombosis are common when treating either CVD or cardiovascular disease [2, 36].

Laboratory biomarkers of the pathophysiological changes in CVD have been identified, with MMP-9, fibronectin, and vascular endothelial growth factor (VEGF) levels showing a positive correlation with clinical severity (i.e., CEAP stage) [37].

### Treatment

Patients with CVD are advised to make lifestyle changes that may reduce the severity of CVD (e.g., weight loss and exercise) [12, 38]. In addition to lifestyle changes, recommended therapies for CVD include compression stockings or pumps, venoactive drugs, sclerotherapy, and eventually surgical treatment [12–15].

Compression is usually chosen as first-line treatment because it is non-invasive. However, compression stockings can be difficult for some patients (e.g., those with a high body mass index) to use, and compliance tends to be poor because of the cost or discomfort related to heat, or because of the occurrence of side effects such as itching [39]. Venoactive drugs include mesoglycan, flavonoids, sulodexide, and others [13, 21]. An Italian consensus statement on the management of CVD specifically recommends pharmacological treatments with anti-inflammatory and endothelial repair properties [12, 38].

### Hemorrhoidal disease

Hemorrhoids are vascular cushions constituting a normal part of the anorectum and are present in all persons; however, most people are unaware of their hemorrhoids until they develop symptomatic HD [17, 40, 41]. Hemorrhoids consist of a plexus of arterioles and venules connected by direct anastomoses (sinusoids), all supported by an epithelial and connective tissue framework that holds the cushion to the muscle of Treitz [42]. The physiological role of the hemorrhoidal plexus is to regulate continence at rest (by maintaining resting anal pressure) and assist with the defecation mechanism, facilitating continence while also protecting the anal sphincters from injury during defecation [40, 41]. Approximately 15% of resting anal pressure is maintained by the hemorrhoidal plexus, with the remaining pressure maintained by the internal and external anal sphincter [43].

Enlarged hemorrhoids may be asymptomatic [17], or they may be associated with itching, discomfort, rectal bleeding, swelling, discharge, pain, or distal displacement of the hemorrhoid through the anus [4, 10]. The severity of HD is commonly graded using the Goligher classification, based mainly on the extent of prolapse (Supplementary Table S2) [44]. Indeed, this classification is based only on fair interobserver agreement, and has been criticized for relying too much on whether a prolapse can be manually reduced and for not reflecting the symptoms that determine treatment [45]. A range of other classification systems have been proposed, based on the size and morphology of the HD, presence of other features (e.g., skin tags, bleeding, edema, thrombosis), and anal sphincter tone [46, 47]. However, no other single classification system has yet replaced the Goligher classification in routine clinical use.

Constipation is an important risk factor for HD because it can increase intra-abdominal pressure, which in turn can affect venous return [10]. Passage of hard stool can also increase shear forces across the hemorrhoidal cushions. Dietary risk factors may include low dietary fiber intake, alcohol consumption, and spicy foods [10]. Pregnancy can have a similar effect on intra-abdominal pressure and hemorrhoidal congestion. Of note, individuals with HD have a high prevalence of CVD [4], suggesting that the two conditions share common etiology or genetic propensity.

### Pathophysiology

The etiopathogenesis of HD is multifactorial and characterized by marked venous dilation, degeneration of the supporting fibroelastic network, and vascular thrombosis [10, 41]. The risk of hemorrhoid dilatation is high because the superior rectal vein and middle rectal vein have no valves to prevent retrograde blood flow [42]. The functional integrity of the fibroelastic network can be compromised by structural or functional changes in the muscle of Treitz (from repeated strained defecations), as well as deterioration in the quality of collagen with increasing age. This problem is exacerbated by increased tone in the internal anal sphincter, which further prevents venous drainage from the sinusoids and exacerbates the congestion [42].

Patients with hemorrhoids have elevated levels of MMP-2 and MMP-9, both of which degrade elastic fibers and cause tissue remodeling, including fibrosis and neovascularization [42]. Similar to the relationship between disease severity and MMP levels in CVD patients, a more severe Goligher grade is associated with increased MMP-9 levels in HD [11]. Additional potential biomarkers of hemorrhoids are other MMPs (specifically MMP-1 and MMP-3 in grade I and II disease, and MMP-3, -7, and -8 in grade III) and neutrophil gelatinase-associated lipocalin [11].

Pathology studies indicate that there is evidence of both acute and chronic inflammatory responses in diseased hemorrhoidal tissue, with elevated expression of endothelial growth factor receptor (EGFR), indicating a wound healing response [48].

### Treatment

Patients with HD are advised to drink plenty of water and consume sufficient fiber to avoid constipation and minimize straining during defecation [17, 49]. In addition to dietary modifications, topical treatments (e.g., glucocorticoids, vasoconstrictors, or analgesics) may help to relieve symptoms [17, 49]. Oral phlebotonics for HD include some of those also used to treat CVD, such as flavonoids, diosmins, rutosides, plant extracts, and calcium dobesilate [18].

A consensus statement from the Italian Society of Colorectal Surgery recommends anti-inflammatory agents or local steroids as first-line therapy [16]; however, in clinical practice, flavonoids and increased fiber intake are more commonly used to treat patients with HD [50]. Interventions for Goligher grade 1–3 hemorrhoids include office-based procedures, such as ligation, sclerotherapy, infrared coagulation, cryotherapy, laser therapy, or radiofrequency ablation [51, 52]. In patients with thrombosed external hemorrhoids, topical vasodilators such as nifedipine or glyceryl trinitrate can relieve pain and reduce the size of the hemorrhoid [53]. When these treatments are ineffective or when hemorrhoids are high grade and symptomatic, surgical intervention is indicated [10, 16, 49]. Patients awaiting surgery can obtain relief through use of local or systemic anti-inflammatory agents [16]. Thrombosis of mucocutaneous bridges after excisional hemorrhoidectomy can be treated with mesoglycan (because of its antithrombotic and profibrinolytic properties) [19, 20, 49, 54], which may help avoid further surgery and reduce the risk of anal stenosis [55].

It is encouraging that enrollment for a randomized, double-blind, placebo-controlled phase II trial (CHORMES; NCT06101992) to evaluate the use of oral mesoglycan in the acute phase of HD will commence in early 2024 [56]. The aim of the study is to evaluate the efficacy and safety of mesoglycan versus placebo in reducing the symptoms of HD and the impact on HRQoL.

## Mesoglycan

### Mechanism of action

Mesoglycan is a natural GAG preparation, typically composed of heparan sulfate (47.5%), dermatan sulfate (35.5%), slow-moving heparin (8.5%), and small but variable quantities of chondroitin sulfate (8.5%) [21]. The electrophoretically slow-moving type of heparin present in mesoglycan has a lower sulfate:carboxyl ratio and greater anticoagulant activity compared with fast-moving heparin [57].

Mesoglycan is available in oral formulations or as a topically applied medical device (Prisma® Skin), which contains mesoglycan and hyaluronic acid in a water-soluble dressing mounted on an inert polyethylene terephthalate (PET) support material.

Mesoglycan has vascular protective, antithrombotic, and wound healing effects [58–62]. One of the vascular protective effects is to suppress vascular smooth muscle cell proliferation through the mammalian target of rapamycin (mTOR)-mediated signaling pathway, causing AMP-activated protein kinase (AMPK) activation [61]. Mesoglycan also preserves endothelial function by restoring the glycocalyx of endothelial cells. Heparan sulfate, the major component of mesoglycan, has been shown to help regenerate the glycocalyx and restore endothelial function in cellular models of glycocalyx degeneration [63]. A study in patients with type 2 diabetes and peripheral arterial disease demonstrated that mesoglycan inhibits the production of markers of endothelial damage, including MMP-2, MMP-9, soluble E-selection, TNF-α, soluble vascular cell adhesion molecule 1 (s-VCAM-1), and interleukin-6, with statistically significant differences compared with baseline and placebo [64]. These changes were accompanied by clinical improvements, such as a reduction in pain and an improvement in ankle/brachial index and transcutaneous oxygen pressure, indicating that the improvements in endothelial function were clinically significant [64].

**Fig. 2:**
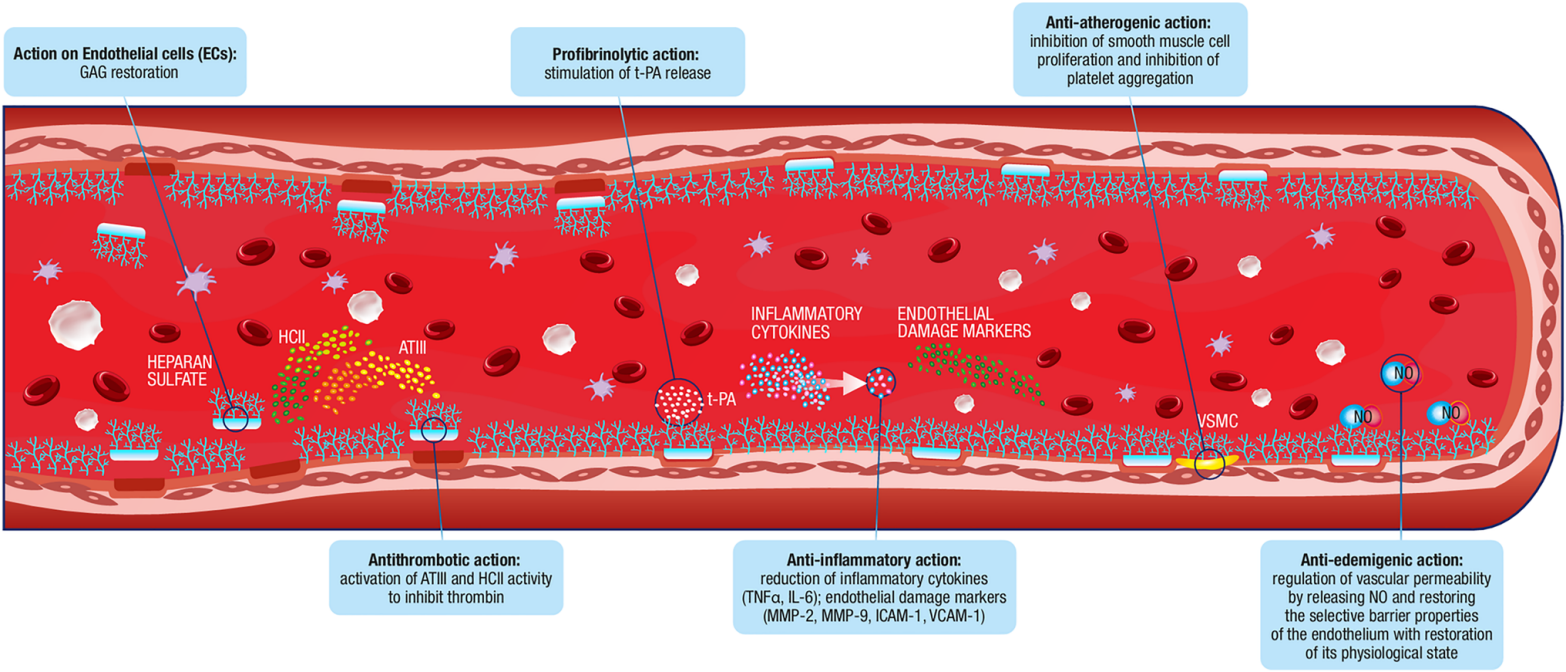
Mechanisms of action of mesoglycan. *ATIII* antithrombin III, *GAG* glycosaminoglycan, *HCII* heparin cofactor II, *t-PA* tissue plasminogen activator, *VSMC* vascular smooth muscle cells

Mesoglycan has antithrombotic and profibrinolytic effects after oral administration in patients with impaired fibrinolysis [62]. The antithrombotic activity of mesoglycan is multifactorial (Fig. 2). The individual components of mesoglycan show antithrombotic activity through interactions with antithrombin III, which inhibits thrombin and factor Xa (heparan sulfate), and with heparin cofactor II, which inhibits thrombin via interactions with heparin and dermatan sulfate [33, 65]. Mesoglycan has also been shown to activate annexin A2 (ANXA2), a cofactor of plasmin generation, which facilitates the cleavage of plasminogen by tissue plasminogen activator, leading to the release of active plasmin, which in turn is able to cleave fibrin and break down clots [57].

In addition to its antithrombotic effects, mesoglycan promotes wound healing via numerous different mechanisms (Fig. 2). Human umbilical endothelial cells exposed to mesoglycan (directly or via the Prisma® Skin device) showed increased endothelial-to-mesenchymal transition, a key step in tissue healing and angiogenesis [58, 66]. Anti-inflammatory effects have also been observed, with suppression of both NO synthesis and nuclear factor (NF)-κB production [66]. In skin cells and fibroblasts, mesoglycan upregulated cell migration in response to injury, thereby accelerating wound healing by enhancing the formation of granulation tissue and re-epithelialization [58]. During this process, mesoglycan amplifies the activation of fibroblasts and endothelial cells through a positive feedback loop that is enhanced by fibroblast growth factor (FGF-2)- and VEGF-mediated signaling [60]. Further, mesoglycan mediates keratinocyte migration and differentiation, and amplifies this process by stimulating the release of ANXA 1 [67].

### Clinical effects in patients with CVD

Mesoglycan has been extensively studied in patients with CVD (Table 2) [68–75].

**Table 2:** Clinical studies of mesoglycan in patients with chronic venous disease

References	Study design	Patient type	*N*	Treatment	Study duration	Main outcomes
Aluigi et al. [70]	Prospective, OL, observational	Varicose veins associated with CVI and/or CVI	202	Mesoglycan 100 mg/day PO	3 months	Significant reduction in CVD symptoms and edema severity vs baseline (*p* < 0.001)
La Marca et al. [74]	Prospective, randomized, OL	CVD with ulcer	40	Topical mesoglycan 1–2 vials/day (*n* = 20) vs plant stimulins (n = 20)	60 days	Ulcer healing rate was 95% with mesoglycan vs 80% with plant stimulins
Arosio et al. [72]	Randomized, DB, placebo controlled	CVD—(C5-C6)	183	Mesoglycan 30 mg/day IM for 3 weeks then 100 mg/day PO (*n* = 92) or placebo (*n* = 91)	24 weeks	Significantly higher rate of ulcer healing (*p* < 0.05) and earlier ulcer healing vs placebo (90 vs 136 days, respectively); significantly better HRQoL (SF-36) in patients with healed vs unhealed ulcers (*p* < 0.05)
Andreozzi, [71]	Retrospective	DVT	83	Mesoglycan 100 mg/day PO	3 years	Low rate of DVT recurrence (cumulative rate of 14.3%)
		(CEAP C_3_, C_4_ or C_6_)	182		3 years	Significant reduction in severity of edema, pain, and disability vs baseline in C_3_ and C_4_ patients (*p* < 0.00001)
Allegra et al. [68]	Prospective, OL, observational	(CEAP C_2_, C_3_ or C_4a_)	1483	Mesoglycan 50 mg BID PO	30 days	Significant improvement in HRQoL (SF-36) vs baseline (*p* < 0.001); significant reduction in CVD severity (edema, hyperpigmentation, or eczema) vs baseline (*p* < 0.0001)
Allegra et al. [69]	Prospective, OL, observational	(CEAP C_2_ or C_3_)	1066	Mesoglycan 50 mg BID PO	2 months	Significant improvement in HRQoL (CIVIQ-20) vs baseline; significant reduction in edema vs baseline
Maresca et al. [75]	Prospective, OL, comparative	Women (CEAP C1 to C_4_)	75	Mesoglycan 50 mg BID (*n* = 37) or standard care (*n* = 38)	90 days	Peak skin microcirculation on LDF significantly increased vs baseline with mesoglycan (*p* = 0.007) and vs standard care (*p* < 0.005)
Kontothanassis et al. [73]	Prospective, OL, observational	(CEAP C_2_ or worse)	316	Mesoglycan 50 mg BID PO	2 months	Low rate of postoperative venous thrombosis (0.8%)

*BID* twice daily, *CEAP* Clinical-Etiological-Anatomical-Pathological [classification system], *CIVIQ-20* 20-item Chronic Venous Insufficiency Quality of Life Questionnaire, *CVD* chronic venous disease, *CVI* chronic venous insufficiency, *DB* double blind, *DVT* deep vein thrombosis, *HRQoL* health-related quality of life, *IM* intramuscular, *LDF* laser Doppler flow, *NR* not reported, *OL* open label, *PO* orally, *SF-36* Short Form 36

Both prospective and retrospective studies have demonstrated a reduction in CVD severity during treatment with oral mesoglycan 100 mg/day [68–71, 75]. For example, a retrospective analysis in 182 patients receiving mesoglycan 100 mg/day demonstrated a significant reduction in disease severity (i.e., edema, pain, and disability) compared with baseline [71]. The largest of the observational studies were two open-label, observational studies in patients with CEAP class C_2_, C_3_, or C_4a_ CVD, one with 1483 patients [68] and the other with 1066 patients [69]. Oral mesoglycan 50 mg twice daily for 1 or 2 months significantly reduced the severity of CVD signs and symptoms, including edema, and improved HRQoL compared with baseline in these two studies [68, 69]. Both studies showed consistent results with regard to HRQoL, whether this was measured using a generic (i.e., the Short Form-36 [SF-36] health survey) or a disease-specific (i.e., the Chronic Venous Disease Quality of Life Questionnaire) tool [68, 69]. It is notable that these two studies assessed patients at 1–4 months after completion of mesoglycan treatment and noted that the symptomatic and HRQoL benefits were maintained after drug withdrawal [68, 69]. These data indicate beneficial effects of mesoglycan on the underlying disease process. This is supported by the results of a comparative study in women with CVD of CEAP class C_1_–C_4_ [73]. In this study, women treated with mesoglycan 100 mg/day for 90 days showed significant improvements in the function of skin microvessels (assessed by laser Doppler flow) compared with standard care (*p* < 0.005) [75].

Clinical data indicate that oral mesoglycan 100 mg/day is associated with a low rate of venous thrombosis in patients with CVD, including in the postoperative setting, although the two studies that examined this outcome did not have control arms, making it difficult to draw conclusions regarding this effect [71, 73].

Two randomized studies have investigated the effects of mesoglycan in patients with CVD and venous ulcers. The first, an open-label study, which compared topical mesoglycan (1–2 vials/day) with topical plant stimulins, found a higher ulcer healing rate with mesoglycan (95%) versus the control arm (80%) [74]. The second was a randomized, placebo-controlled, double-blind study, in which patients received 3 weeks of intramuscular mesoglycan injections (30 mg/day), followed by oral mesoglycan 100 mg/day or placebo for a total of 24 weeks [72]. In the double-blind study, the rate of ulcer healing was significantly higher with mesoglycan versus placebo at the end of treatment (97% vs 82%; *p* < 0.05), with a shorter median time to ulcer healing in the mesoglycan group than in the placebo group (64 vs 70 days). No bleeding was observed in the mesoglycan group, whereas rectal bleeding was observed in the placebo group. In addition, patients with healed ulcers had significantly better HRQoL compared with those with unhealed ulcers [72].

The ongoing METRO study (NCT03428711) is currently investigating the efficacy and safety of oral mesoglycan for the secondary prevention of venous thromboembolic (VTE) complications in 650 patients with SVT who have completed acute therapy (subcutaneous fondaparinux 2.5 mg once daily for 45 days). Participants are randomized to oral mesoglycan 50 mg twice daily or matching placebo for 12 months and then followed for a further 12 months without treatment. Participants and investigators/outcome assessors are all blinded to the patient’s treatment allocation. The primary end point is the cumulative occurrence of the first event (with instrumental confirmation) of recurrence or extension of SVT (asymptomatic or symptomatic), new proximal or distal DVT (symptomatic or asymptomatic) or pulmonary embolism (fatal or symptomatic nonfatal) over 12 months. The study is expected to finish on 31 December 2024. The METRO study will provide important information about the efficacy and safety of mesoglycan in preventing SVT recurrence and/or VTE events after the completion of acute-phase therapy; about the long-term protective effect of mesoglycan compared with placebo on the venous wall; and about the natural history of SVT over a 2-year follow-up period in patients randomized to placebo.

### Clinical effects in patients with HD

Three studies have investigated the use of mesoglycan treatment in patients with HD (Table 3) [19, 20, 76].

**Table 3:** Clinical studies of mesoglycan in patients with hemorrhoidal disease

References	Study design	Patient type	*N*	Mesoglycan treatment	Study duration	Main outcomes
Saggioro et al. [76]	Prospective, randomized, OL	Acute grade II and III HD	71 (36 mesoglycan, 35 bilberry extract)	24 mg TID PO	28 days	Significantly greater reductions in severity of pain, sensation of pressure, pruritus, edema, inflammation, and rectal bleeding with mesoglycan vs bilberry extract
Gallo et al. [20]	Prospective, Pilot	After excisional hemorrhoidectomy in grade III and IV HD	101 (54 mesoglycan, 47 standard care^a^)	30 mg/day IM for 5 days, then 50 mg BID PO for 30 days	40 days	Mesoglycan significantly reduced pain during rectal examination (*p* = 0.033) and time to return to work/normal activities (*p* = 0.009) vs standard care^a^; significantly (*p* < 0.001) lower rate of thrombosis on postoperative days 7–10 with mesoglycan vs standard care^a^
Gallo et al. [19]	Retrospective	After excisional hemorrhoidectomy in grade III and IV HD	398 (206 mesoglycan, 192 standard care^a^)	30 mg/day IM for 5 days, then 50 mg BID PO for 30 days	6 weeks	Mesoglycan significantly reduced pain at rest (all *p* < 0.0001), after defecation (all *p* < 0.0001) and during examination (*p* < 0.0001, *p* < 0.0001, and *p* = 0.003, respectively) vs standard care^a^ after 1, 3, and 6 weeks; mesoglycan significantly improved postoperative activities, autonomy, driving, and return to work vs standard care^a^ after 1 (*p* = 0.016), 3 (*p* = 0.002) and 6 (*p* = 0.007) weeks

*BID* twice daily, *HD* hemorrhoidal disease, *IM* intramuscular, *OL* open label, *PO* orally, *TID* three times daily

^a^Oral dose of ketorolac 10 mg every 4–6 h, not exceeding 40 mg/day and not exceeding 5 days + stool softeners

The first of these was a randomized, open-label study that compared oral mesoglycan 24 mg three times daily with a bilberry extract preparation 160 mg twice daily [76]. After 28 days, the severity of HD signs and symptoms had decreased in both groups, but by a significantly greater extent in patients receiving mesoglycan compared with those receiving bilberry extract (*p* < 0.001). The number of rectal bleeding episodes also decreased significantly more with mesoglycan (from 6.2 episodes in the 2 weeks prior to treatment to 1.1 episodes during days 14–28) than with bilberry extract (from 6.4 episodes to 2.5 episodes, respectively; *p* < 0.05) [76].

Two recent studies by Gallo and colleagues have examined the impact of adding mesoglycan to standard postoperative care in patients who have undergone excisional hemorrhoidectomy [19, 20]. Patients undergoing this procedure frequently experience severe postoperative pain [77, 78], but Gallo and colleagues hypothesized that mesoglycan would aid recovery after hemorrhoidectomy by reducing edema in the mucocutaneous bridges and preventing the development of local thrombosis [19, 20]. First, they conducted a pilot study in 101 patients undergoing excisional hemorrhoidectomy with diathermy [20], in which patients were assigned to mesoglycan (applied topically for the first 5 days and then taken orally for 30 days) and/or ketorolac for 5 days postoperatively. The incidence of postoperative thrombosis (*p* < 0.001) and pain associated with rectal examination (*p* = 0.033) at 7–10 days after surgery was significantly lower with mesoglycan than with ketorolac, and patients in the mesoglycan group were able to return to work earlier compared with patients in the ketorolac group (*p* = 0.009). The incidence of bleeding was higher in the mesoglycan than the ketorolac group, but the between-group difference was not statistically significant (*p* = 0.488) [20].

The Mesoglycan for pain control after open excisional HAEMOrrhoidectomy (MeHAEMO) study was a retrospective observational study in 398 patients who underwent surgery at 16 colorectal referral centers [19]. All patients received standard postoperative therapy with ketorolac for the first 5 days, but 206 patients also received mesoglycan as administered in the pilot study described above. Patients were assessed on the first postoperative day, then 1, 3 and 6 weeks after discharge. The incidence of postoperative thrombosis was significantly lower in the mesoglycan than the control group at 1 week (6.3% vs 12.5%, respectively; *p* < 0.05) and 3 weeks (3.3% vs 10.4%; *p* = 0.005) after surgery, while a higher proportion of patients in the mesoglycan group than the control group had returned to work at 1 week (17.3% vs 6.8%; *p* = 0.016), 3 weeks (60.7% vs 47.3%; *p* = 0.002), and 6 weeks (72.3% vs 62.5%; *p* = 0.007) [19]. Pain scores for pain at rest, after defecation, and after anorectal digital examination were significantly lower in the mesoglycan than the control group at 1, 3, and 6 weeks after discharge (*p* ≤ 0.003). Scores for both physical and mental domains of HRQoL (measured by SF-36) were also significantly higher in the mesoglycan than the control group (*p* < 0.0001), and there were no significant between-group differences in the rate of bleeding [19].

## Conclusions

CVD and HD share many common risk factors and pathophysiological features, and often occur concurrently. In addition to lifestyle and dietary modifications, pharmacological therapy for both conditions should include agents that have antithrombotic and anti-inflammatory properties that help to reverse or repair the underlying pathophysiological processes. Mesoglycan contains a mixture of GAGs that target the underlying disease processes in CVD and HD, making it a reasonable treatment choice for patients with these conditions. While much of the evidence for mesoglycan comes from observational and open-label studies, which may be associated with limitations such as bias, confounding, and issues with validity, the efficacy of mesoglycan has been demonstrated in patients with CVD or HD, where the clinical effects of treatment (i.e., wound healing, improvement of signs and symptoms, and reduction of the incidence of thrombosis) are consistent with the agent’s known mechanisms of action.

### Supplementary Information

Below is the link to the electronic supplementary material.

Supplementary file1 (DOCX 27 KB)

## Data Availability

Data sharing is not applicable to this article as no new data were created or analyzed in this study.
