# Supplementary material for: Clinical evidence and rationale of mesoglycan to treat chronic venous disease and hemorrhoidal disease: a narrative review
Source: Updates Surg. 2024 Feb 14;76(2):423–34. doi: 10.1007/s13304-024-01776-9 (PMC10995001; doi:10.1007/s13304-024-01776-9)
Supplement: Supplementary file 1 — Supplementary file1 (DOCX 27 KB) [file 13304_2024_1776_MOESM1_ESM.docx]

# Supplementary materials

## Supplementary Table S1.

Summary of the clinical [C] classification of CEAP in the 2020 revision [22]. Reprinted from the Journal of Vascular Surgery: Venous and Lymphatic Disorders, Vol 8, Lurie F, Passman M, Meisner M, Dalsing M, Masuda E, Welch H, et al., The 2020 update of the CEAP classification system and reporting standards, Pages 342-352, Copyright (2020), with permission from Elsevier.

| **C class** | **Description** |
| --- | --- |
| C_0_ | No visible or palpable signs of venous disease |
| C_1_ | Telangiectasias or reticular veins |
| C_2_ | Varicose veins |
| C_2r_ | Recurrent varicose veins |
| C_3_ | Edema |
| C_4_ | Changes in skin and subcutaneous tissue secondary to CVD |
| C_4a_ | Pigmentation or eczema |
| C_4b_ | Lipodermatosclerosis or atrophie blanche |
| C_4c_ | Corona phlebectatica |
| C_5_ | Healed venous ulcer |
| C_6_ | Active venous ulcer |
| C_6r_ | Recurrent active venous ulcer |

*CVD* chronic venous disease

## Supplementary Table S2.

Goligher classification system for the severity of hemorrhoids [40].

| **Grade** | **Description** |
| --- | --- |
| I | Hemorrhoidal engorgement above the dentate line but no prolapse |
| II | Hemorrhoidal tissue prolapses below the dentate line during straining but spontaneously reverts |
| III | Hemorrhoidal tissue prolapses below the dentate line during straining and requires manual intervention to return to the rectum; itching, soiling, mucous discharge and swelling may be present |
| IV | Prolapsed hemorrhoids are evident on examination and cannot be reduced manually; inflammatory changes, maceration, mucosal atrophy and ulceration may be present |
